# Retraction: uPAR/Cathepsin B Overexpression Reverse Angiogenesis by Rescuing FAK Phosphorylation in uPAR/Cathepsin B Down Regulated Meningioma

**DOI:** 10.1371/journal.pone.0228681

**Published:** 2020-01-29

**Authors:** The PLOS ONE Editors

After this article [1] was published, concerns were raised about the following results:

- Fig 1A, CH-157MN, 5gy panel appears similar to Fig 4C, CH-157MN, +IR, pUC+FLpU/C panel.
- Fig 1D, IOMM-Lee, -IR, pSV panel appears similar to Fig 4C, IOMM-Lee, -IR, pUC+FLpU/C panel.
- Fig 2C, the -IR images for pUC and pU2 appear to report overlapping data.
- Fig 2D, Ang-1, +IR panel, lanes 2 and 3 (pUC, pU2) appear similar.
- Figs 2A, 2D, 2E, 3A, 4A, 5A, 6A, S1A: there are image splicing lines between the -IR and +IR data in each figure and it is unclear whether the plus and minus treatment results were obtained in the same experiments using the same blots and exposures.
- Fig 2E
  ○ Ang-1(60): There appears to be a vertical discontinuity after lane 4 (+IR, pSV).
  ○ VEGF(15): There appear to be similarities between data in lanes 5 and 6 (+IR data for pUC and pU2), although the main bands appear at different vertical positions.
- Fig 3A
  ○ IOMM-Lee, p-FAK (Y925)(125): There appear to be vertical discontinuities before the bands in lanes 3 and 6; lanes 5 and 6 appear similar; and the background appears to be of a different intensity in lane 5 versus lane 6.
  ○ IOMM-Lee, GAPDH(37): Lanes 1 and 4 appear similar.
- Fig 3C: There appear to be repeated image elements in the p-FAK(Y925) panels for pSV, pSV+IR, pU2, and pU2+IR, and the images in the DAPI, p-FAK(Y925), and Merged panels do not align in some cases.
- Fig 4C: There appear to be repeated image elements in the in-vivo, +IR, pUC+FLpU/C panel.
- The in-vivo GAPDH panels in Figs 4A and 5A appear similar, although with different aspect ratios.
- Data shown in Fig 5A for in-vivo, -IR, Ang-1 (60), appear similar to data reported for a different experiment (DAOY, Bax lanes 1–3) in Fig 5C of [2]. Also, there appears to be a vertical discontinuity after lane 1 in this panel of [1].
- Fig 5A, IOMM-Lee, -IR, Ang-2(70) panel appears similar to the Fig 6A in-vivo, +IR, MEK1(45) panel.
- Fig 5A in-vivo, +IR, Ang-1(60) panel appears similar to the Fig 6A in-vivo, +IR, Y925(125) panel.
- Lane 1 of Fig 5A, IOMM-Lee, Ang-2(70) and lane 1 of Fig 6A, in-vivo, +IR, MEK1(45) appear similar to data reported for a different experiment (D283, Bcl2, lane 4) in Fig 5C of [2]
- Fig S1A, in vivo FAK panel, data appear similar in lanes 2 and 3 and in lanes 5 and 6, and there appears to be a vertical discontinuity before lane 6. Also, Fig S1A includes only a single GAPDH control panel although the figure reports three different experiments (IOMM-Lee, CH-157MN, in vivo).

The above concerns call into question the integrity and reliability of the published results, and so the *PLOS ONE* Editors retract this article.

We attempted to contact the article’s authors to discuss these issues, request the underlying data, and notify them of the retraction decision, but the authors either did not reply or could not be reached.

The IOMM-Lee, Ang-2(70) panel in Fig 5A, the in-vivo, -IR, Ang-1 panel in Fig 5A, and the in-vivo, +IR, MEK1 panel in Fig 6A [1] appear to report material from [2], published in 2011 [Spandidos Publications] which are not offered under a CC-BY license. These panels are therefore excluded from the *PLOS ONE* article’s [1] license. At the time of retraction, the article [1] was republished to note this exclusion in the Figs 5 and 6 legends and the article’s copyright statement.

## References

[1] Gupta R, Nalla AK, Gogineni VR, Chetty C, Bhoopathi P, Klopfenstein JD, et al (2011) uPAR/Cathepsin B Overexpression Reverse Angiogenesis by Rescuing FAK Phosphorylation in uPAR/Cathepsin B Down Regulated Meningioma. PLoS ONE 6(2): e17123 10.1371/journal.pone.0017123 21347260 PMC3037969

[2] Gupta R, Chetty C, Bhoopathi P, Lakka S, Mohanam S, Rao JS, Dinh DE. (2011) Downregulation of uPA/uPAR inhibits intermittent hypoxia-induced epithelial-mesenchymal transition (EMT) in DAOY and D283 medulloblastoma cells. Int J Oncol. 38(3):733–44. 10.3892/ijo.2010.883 21181094

